# Identification of a short sequence in the HCMV terminase pUL56 essential for interaction with pUL89 subunit

**DOI:** 10.1038/s41598-017-09469-7

**Published:** 2017-08-18

**Authors:** G. Ligat, C. Jacquet, S. Chou, A. Couvreux, S. Alain, S. Hantz

**Affiliations:** 1 Université Limoges, INSERM, CHU Limoges, UMR 1092 Limoges, France; 2 CHU Limoges, Laboratoire de Bactériologie-Virologie-Hygiène, National Reference Center for Cytomegaloviruses (NRC), Limoges, France; 3 Division of Infectious Diseases, Oregon Health and Science University, Portland, Oregon, USA and Research Service, VA Portland Health Care System, Portland, Oregon USA

## Abstract

The human cytomegalovirus (HCMV) terminase complex consists of several components acting together to cleave viral DNA into unit length genomes and translocate them into capsids, a critical process in the production of infectious virions subsequent to DNA replication. Previous studies suggest that the carboxyl-terminal portion of the pUL56 subunit interacts with the pUL89 subunit. However, the specific interacting residues of pUL56 remain unknown. We identified a conserved sequence in the C-terminal moiety of pUL56 (_671_WMVVKYMGFF_680_). Overrepresentation of conserved aromatic amino acids through 20 herpesviruses homologues of pUL56 suggests an involvement of this short peptide into the interaction between the larger pUL56 terminase subunit and the smaller pUL89 subunit. Use of Alpha technology highlighted an interaction between pUL56 and pUL89 driven through the peptide _671_WMVVKYMGFF_680_. A deletion of these residues blocks viral replication. We hypothesize that it is the consequence of the disruption of the pUL56-pUL89 interaction. These results show that this motif is essential for HCMV replication and could be a target for development of new small antiviral drugs or peptidomimetics.

## Introduction

Human cytomegalovirus (HCMV), a beta herpesvirus, can cause serious diseases in immunocompromised patients. Current antiviral inhibitors (ganciclovir, cidofovir and foscarnet) all target the viral DNA polymerase. They have adverse effects and prolonged treatment can select for drug resistance mutations either in the viral polymerase pUL54, the kinase pUL97 or both of them^1, 2^. Thus, we need new drugs targeting others stages of replication. The terminase complex is highly specific for HCMV, has no counterpart in the human organism, and thus represents a target of choice for new antivirals development. This has been confirmed by the recent development of letermovir in the transplant setting^3, 4^.

DNA packaging process requires several proteins such as pUL56 and pUL89, the large and small terminase subunits, respectively. Recently, four additional proteins were shown to be also implicated in this process, namely, pUL51, pUL52, pUL77, pUL93^5–10^. This process is driven by specific interactions of protein-DNA and protein-protein to cleave and package unit length genomic DNA into an empty capsid.

Evidence suggests that the large subunit pUL56 has a crucial role in DNA cleavage/packaging, containing many of the functional sites required for this process like interaction with the portal protein pUL104, endonuclease activity, and more interestingly an ATP-binding site (amino acids 709 to 723)^11^. Although the association between pUL56 and pUL89 has already been reported, the residues of pUL56 involved in the terminase complex integrity are still unknown^12, 13^. Nevertheless, co-immunoprecipitation experiments showing an interaction between the C-terminal half of pUL56 (pUL56-Cter) and pUL89 were confirmed by other results^12, 13^.

Because knowledge of terminase functional and interaction domains is important both for the development of drugs targeting the DNA packaging stage and for the improvement of existing ones such as letermovir, the aim of the current study is to identify a minimum peptide of pUL56 with a putative key role in its interaction with pUL89. Sequence alignments encouraged us to focus on the putative involvement of one part of the pUL56 sequence into its interface with pUL89. BAC mutagenesis and Alpha technology using purified proteins subsequently validated that the aromatic rich peptide _671_WMVVKYMGFF_680_ pUL56(671-680) in the C-terminal of pUL56 is involved in interaction with pUL89. These results could contribute for development of new antiviral drugs, peptides or antibodies against HCMV.

## Results

### A putative conserved protein interface in pUL56 subunit

**Figure 1:**
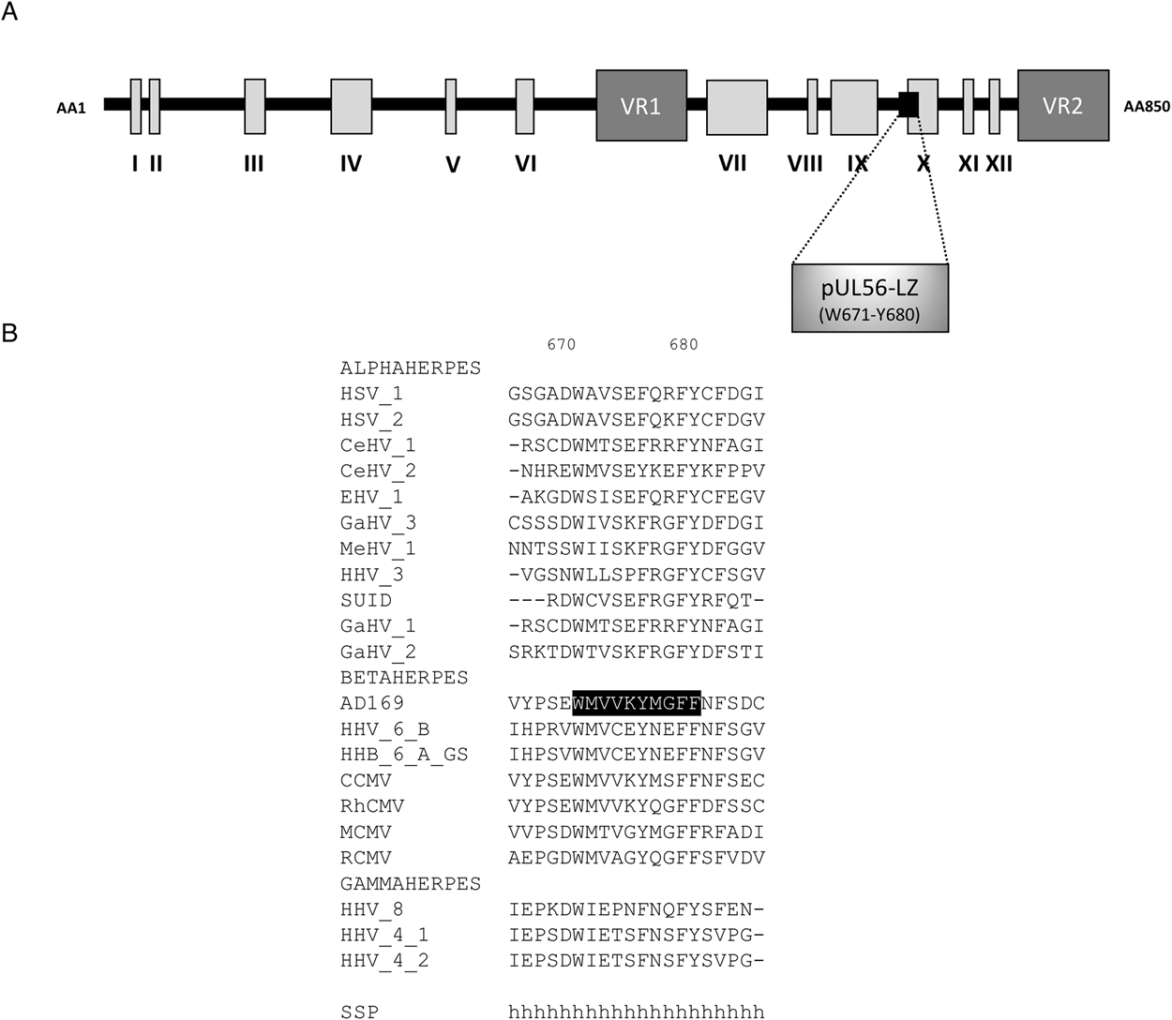
(**A**) Structure of HCMV terminase subunit pUL56 with a putative leucine zipper pattern annotated as pUL56-LZ^22^. (**B**) Sequences alignment of conserved regions from 21 herpesviruses. Sequence numbering is consistent with that of AD169 residues. Key residues are highlighted as white letters on a black background. SSP: Secondary structure prediction of pUL56-LZ (h = α helix).

Selection of a potent pUL56 fragment for pUL89 interaction was supported by three hints. First, based on the sequences alignment of pUL56 with 20 herpesviruses homologues, the peptide _671_WMVVKYMGFF_680_ pUL56(671-680) seems to be broadly conserved in betaherpesviruses proteins, which supported a major role either in function or structure of pUL56. Secondly, as shown in Fig. 1, its secondary structure is predicted as an alpha helix. Previous studies demonstrated that the peptide pUL89(580-600) implicated in the pUL56-pUL89 interface^13^ adopts an alpha helix secondary structure^14, 15^. Moreover, wide protein-protein interfaces analyses revealed a preferential interaction of an helix of one protein with one of its counterpart^16, 17^.

Thirdly, pUL56(671-680) is within the C-terminal part previously described to be sufficient for interaction with pUL89^12^. Interestingly, this motif belongs to the pUL56 region carrying the ATP binding site. As a parallel, pUL89(580-600) is enclosed into the endonuclease domain of pUL89^15^. Both activities, ATPase of pUL56 on the one hand and nuclease of pUL89 on the other hand are dependent on the association between the two terminase subunits^12, 18^. Taken together, these observations make pUL56(671-680) a good candidate to interact with pUL89.

### A deletion or targeted mutations of _671_WMVVKYMGFF_680_ pUL56 domain affects viral replication in MRC-5 cells

To evaluate the importance of the pUL56 predicted domain for viral replication, we produced by “en passant” mutagenesis recombinant EGFP-virus with complete deletion of *UL56*(671-680) or point mutations in this sequence. Analysis of HCMV genome confirmed that *UL56* sequence does not encode a gene on the other strand^19^. Thus, mutations in the virus are silent on the other strand and thus cannot impact the function of another gene expressed from the other strand. To ensure that no other mutations that could have a negative impact on viral replication was introduced in the BAC backbone during the manipulations, we performed NGS sequencing on both the original BAC and the mutants. The deletion was found in 100% of the mutants BAC sequences whereas other SNPs were located in genes non essential for viral replication and represent less than 30% of the sequences both in the original BAC and in the mutants.

**Figure 2:**
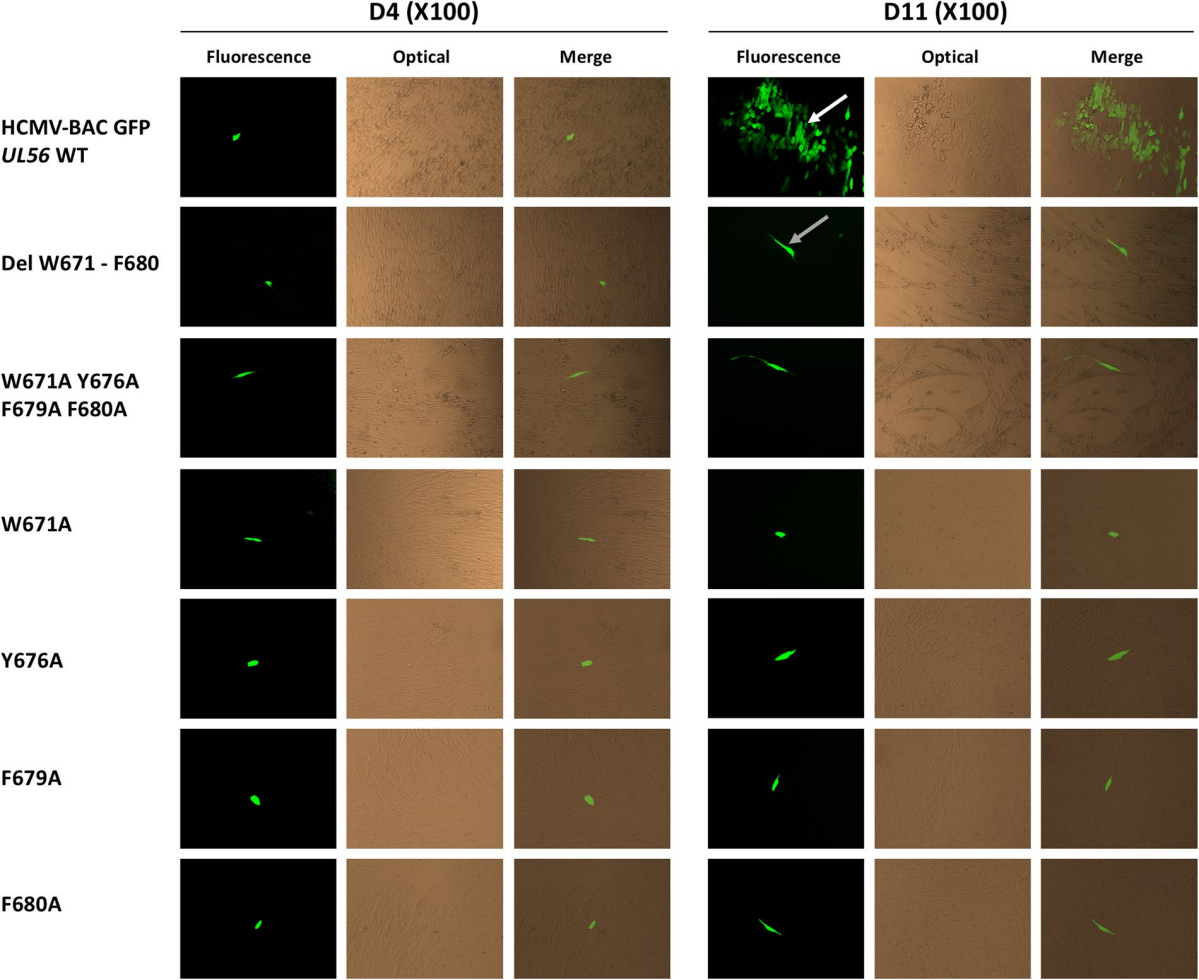
Plaque formation assay in MRC-5 cells after transfection of HCMV-BAC GFP *UL56* WT (AD169) or recombinant virus strains with a deletion, a combination of mutations or a single mutation. Green fluorescent foci (white arrow) were observed with the wild-type HCMV-BAC GFP *UL56* and single infected cells (grey arrow) were observed with the other recombinants viruses. Eleven days after transfection of human fibroblasts, we observed no cytopathic effect for either the mutant with a deletion of _671_WMVVKYMGFF_680_ sequence, or a combination of mutations or single mutations. These mutations dramatically impaired viral replication and propagation in cell-culture.

**Figure 3:**
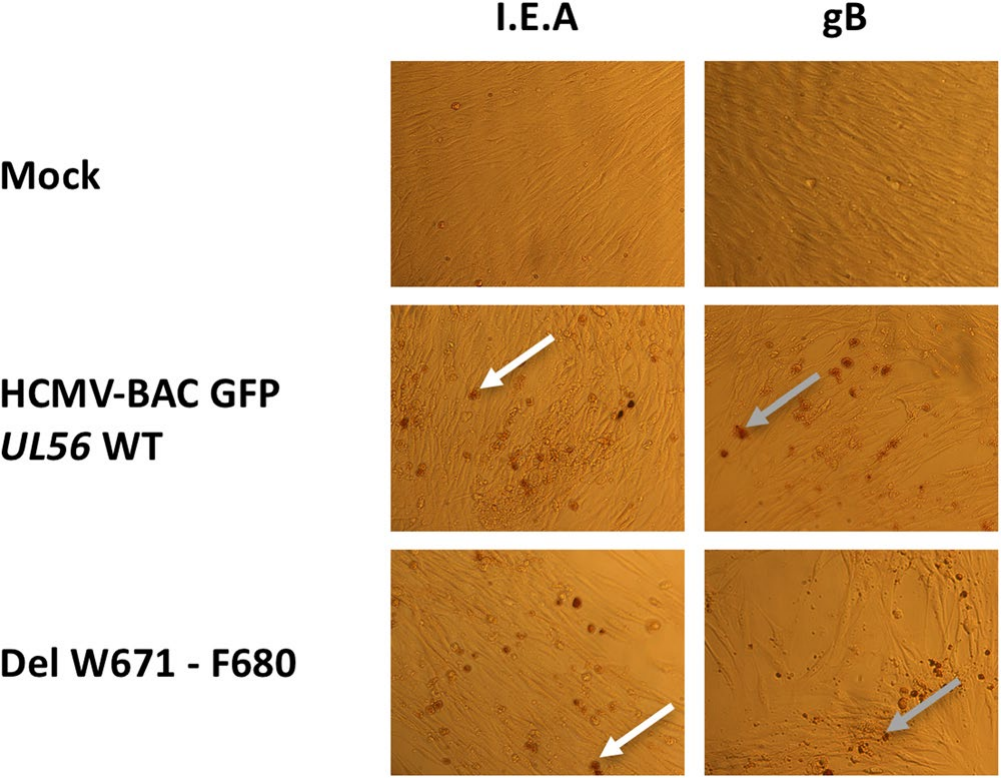
Fragment _671_WMVVKYMGFF_680_ of pUL56 is not required for viral gene expression. MRC-5 were transfected with HCMV-BAC WT or the mutant with a deletion of _671_WMVVKYMGFF_680_ sequence. Five days after transfection, immunostaining was performed for immediate early (I.E.A) (white arrow) and late (gB) (grey arrow) viral proteins.

Unlike the wild-type HCMV-BAC, eleven days after the transfection of human fibroblasts (MRC-5 cells), we observed no foci of cytopathic effect for the mutant which has a deletion of _671_WMVVKYMGFF_680_ sequence (Fig. 2). This deletion dramatically impaired viral replication and propagation in cell-culture. In the same way, recombinant EGFP-viruses with a single or a combination of mutations among W671A, Y676A, F679A and F680A do not produce progeny virion as well. These residues were selected for mutagenesis because they are perfectly or for the less highly conserved (i.e. replaced by another aromatic amino acid) among all the 20 herpesviruses homologues of pUL56 (Fig. 1). To check if these deletion or mutations may disrupt another step of the viral replication, immunostaining assays were performed to detect proteins produced at immediate early and late stages of viral cycle (IE and late proteins). Expression of immediate early (IEA) and late (gB) viral genes were detected indicating that mutations have no impact on viral gene expression (Fig. 3). Therefore W671, Y676, F679 and F680 within pUL56(671-580) are critical amino acids for viral replication.

### pUL56(671-680) is necessary for pUL89 association

HEK293 were transfected with SC784 and pCI-neo His-89 expression plasmids and protein-protein interactions were carried out by the Alpha assay. This technology represents a powerful method to highlight protein-protein interactions^20, 21^. Since we have no virion production for mutant viruses, we chose to study *in vitro* biochemical interactions after protein overexpression in HEK cells which allow introduction of tags (HA and His) for the Alpha assay.

**Figure 4:**
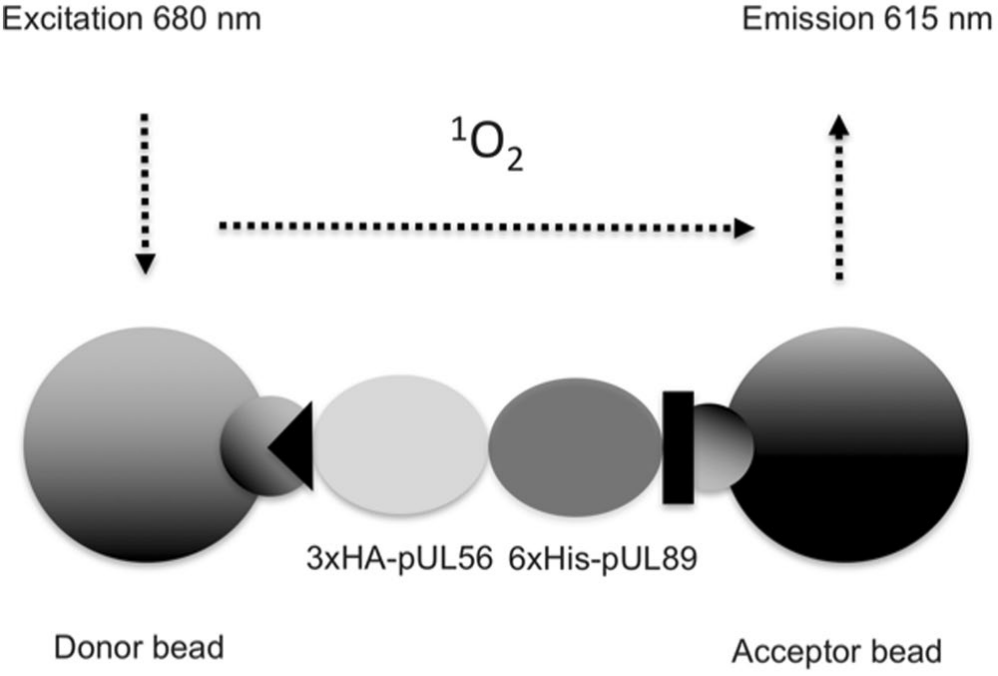
Scheme of an Alpha protein-protein interaction assay, using HA-coated Donor beads, His-coated Acceptor beads, 3xHA-pUL56, 6x His-pUL89. In an Alpha interaction assay, one protein is captured on the Donor beads, and the other protein is captured on the Acceptor beads. In case of interaction, the Donor bead is brought into proximity of the Acceptor bead, and excitation of the Donor bead will result in signal generation dependent on the presence of an interaction.

**Figure 5:**
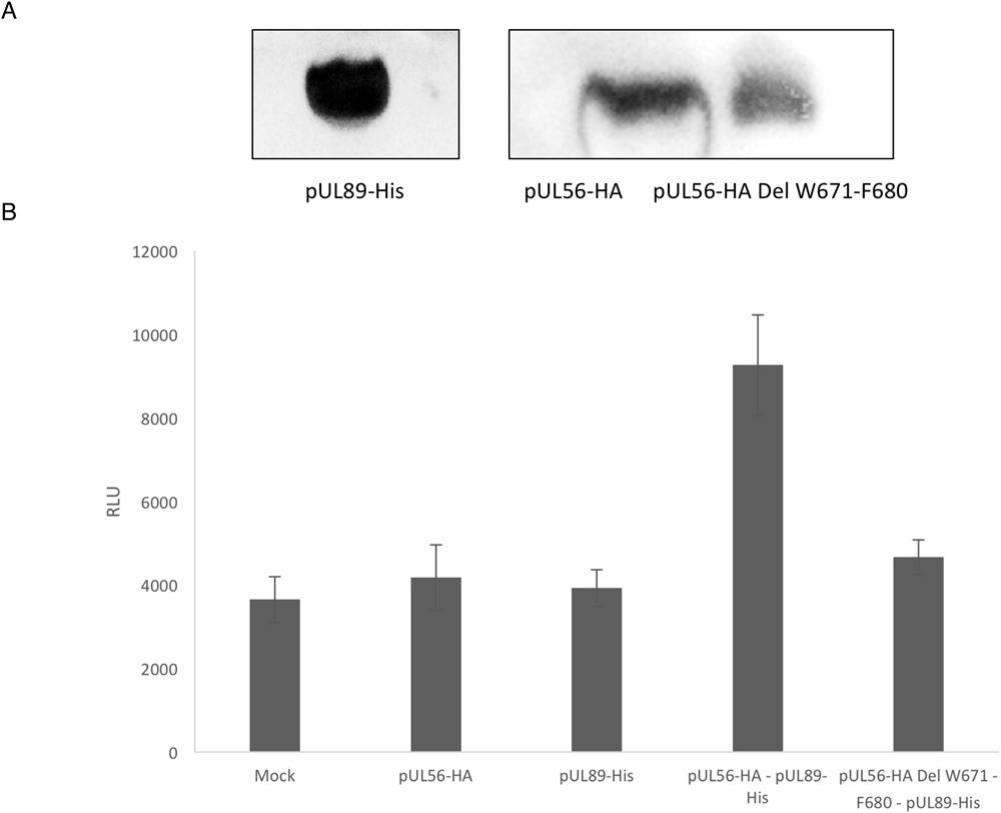
Determination of pUL56 binding domains for the interaction with pUL89. (**A**) Analysis of protein production for alpha assay. Immunoblot was performed using the anti-His antibody for pUL89-His or the anti-HA antibody for pUL56 and pUL56-HA Del W671-F680 and secondary rabbit anti-mouse HRP conjugated antibody. (**B**) Alpha assay results. The Alpha assay for the binding of full-length pUL89 (His-pUL89, 1.5E + 03 nM) was performed with 5E + 02 nM wild-type pUL56 (HA-pUL56) or a deletion mutant of pUL56 (HA-pUL56 Del W671-F680). As a negative control, proteins were used alone and a reaction was performed without proteins (mock). Two measures for each reaction were performed in duplicate.

Alpha assay needs both acceptor and donor beads. For this study, HA-coated Donor beads and His-coated Acceptor were used. A singlet of oxygen diffuses from Donor bead to the Acceptor bead, resulting in light production at 615 nm. In the absence of a specific biological interaction between proteins, singlet molecules produced by the Donor bead cannot be detected beyond 200 nm from the Acceptor bead (Fig. 4). First step consisted in verifying the interaction between pUL56-WT and pUL89-WT as a valuable positive control. Alpha assays with 3xHA-pUL56 and 6xHis-pUL89 results in the production of over 9,000 relative light units (RLU), over two-fold more than negative controls (3xHA-pUL56 or 6x His-pUL89) (Fig. 5). pUL56 depleted of its W671-F680 fragment was in turn soaked with pUL89-WT and their affinity assessed by Alpha analysis. The lack of pUL56(671-680) decreased the interaction signal by 50% which is significant in this assay. These data strongly suggest that _671_WMVVKYMGFF_680_ is necessary for interaction with pUL89.

## Discussion

Protein-protein interactions are essential for several biological pathways such as herpesviruses DNA-packaging. Terminase subunits are proteins forming a hetero-oligomeric complex involved in this process. The HCMV terminase complex is composed of the large subunit pUL56 and the small subunit pUL89. Four additional HCMV proteins, namely, pUL51, pUL52, pUL77, pUL93 contribute also to this process^6–10^. To date, the structural knowledge of herpesviruses terminases is poorly understood including interactions inside its molecular assembly. Previous studies suggest that the large subunit pUL56 has an essential role in this process and contains several functional patterns as a zinc finger domain and a C-terminal nuclear localization signal (NLS)^22, 23^. Although pUL56-Cter is sufficient to interact with pUL89(580-600) subunit^13^, precise moieties of pUL56 constituting the interface against pUL89 are still unknown^12, 13^.

In the present study, we show that deletion of pUL56(671-680) abolishes HCMV replication. We then furtherly checked the impact of this sequence on interaction with pUL89 using Alpha technology. Consistent with our hypothesis, deletion of residues W671 to F680 drastically affects the interaction between pUL56 and pUL89. We propose that the peptide WMVVKYMGFF is crucial for the interaction with pUL89 and thereby for DNA-packaging. Interestingly, this motif is close to the pUL56 region carrying the ATP-binding site (amino acids 709 to 723)^11^. It has been previously demonstrated that the ATPase activity responsible for HCMV DNA translocation into capsids is only associated with pUL56 and is enhanced by up to 30% when pUL56 is associated with pUL89^12^. In this study, we show a close proximity in the pUL56 sequence between interaction site with pUL89 and the ATP-binding site. Moreover, it is important to highlight that interaction locus W671 to F680 is near to the point mutation A662V selected under tomeglovir (Bay38-4766), a non-nucleoside inhibitor of HCMV^24^.

The terminase complex is highly CMV-specific, as no counterpart in mammalian cells exists, and thus represents a promising therapeutic strategy for new antivirals development. This has been confirmed by the recent development of the terminase inhibitor letermovir in the transplant setting^3^. However, its precise site of action in the terminase complex is not yet understood. Clues are offered by a large number of letermovir resistance mutations in *UL56* that have been identified, clustered at *UL56* codons 231–369^25, 26^, and the uncommon selection of *UL89* D344E under letermovir, which combines with *UL56* mutations to increase the overall level of drug resistance. This hints at the possibility of regions of pUL56 and pUL89 that are close to a small molecule drug binding site. Other terminase inhibitors preferentially select for *UL89* mutations, such as D344E for benzimidazole compounds, along with *UL56* mutations at loci such as codons 204 and 662, suggesting yet other possibilities for subunit interactions. A better understanding of all these potential interactions between terminase subunits of HCMV could be valuable when studying the mechanism of action of drugs and the design of new antivirals such as peptidomimetics^27^, antibodies or small molecules that target the interaction domain between these essential viral proteins. Indeed, alteration of protein-protein interaction could be used as a way of inhibition of HCMV replication. A modified peptide based on the WMVVKYMGFF scaffold could serve as molecular target-decoy by interacting with pUL89 and so disrupt the interaction between pUL56 and pUL89. Moreover, we could consider combinations tests of peptides or antibodies with currently available anti-HCMV drugs.

In conclusion, the data from the present study demonstrated that the pUL56 sequence _671_WMVVKYMGFF_680_ is necessary for its interaction with pUL89 and could constitute a good target to suppress this interaction and thus block HCMV replication.

## Materials and Methods

### Identification of conserved patterns and secondary structure prediction

The pUL56 amino acid sequence of reference strain AD169^28^ was aligned with the sequences of 21 homologous proteins from other herpesviruses, as described in Supplementary Table 1. Alignments were performed with Clustal Omega (Ω) multiple sequence alignment (MSA) tool provided by the EMBL-EBI bioinformatics web and programmatic tools framework^29–31^. Secondary structure prediction was carried by Phyre^2^ web portal^32^.

### Cells and bacterial strains

Human fibroblasts MRC-5 (Biomerieux, France) were cultivated at 37 °C in 5% CO_2_ and grown in minimal essential medium (MEM) containing 10% fetal bovine serum with antibiotics. HEK293 (ATCC® CRL-1573™) were cultivated at 37 °C in 5% CO_2_ and grown in minimal essential medium (MEM) containing 10% fetal bovine serum with antibiotics. *E. coli* strain DH5α and Stellar^TM^ (Clontech, USA) were used for cloning procedures. *E. coli* strain GS1783 was used for BAC mutagenesis^33^.

### BAC mutagenesis and reconstitution of mutant viruses

Conserved domains were deleted by “en passant” mutagenesis, a two-step markerless Red recombination system for BAC mutagenesis in *E. coli* strain GS1783. *UL56* point mutations were introduced into an EGFP-expressing HCMV-BAC^33^ to generate several mutants (primers used for mutagenesis are described in Supplementary Table 2). Presence of mutations in *UL56* gene of each virus was confirmed by sequencing prior to transfection. The HCMV-BAC contains an enhanced green fluorescent protein (EGFP) gene in the unique short region and was derived from parental strain pHB5, a BAC-cloned genome of the CMV laboratory strain AD169^33^. The impact of different mutations on viral growth was assessed using transfection of mutated HCMV-BAC into human fibroblasts MRC-5 using liposomal reagent Transfast^TM^ (Promega, USA) following manufacturer’s instructions^34^.

### Library construction and whole-genome DNA sequencing

After HCMV-BAC preparation, amplicons were purified using magnetic beads (Agencourt AMPure XP) and fragmented using the Ion Xpress Plus DNA Fragment Library Preparation kit (Life Technologies). Barcodes adapters were ligated to fragment ends and 250 bp fragments were collected. The library was PCR amplified, then sequenced on the Ion Proton with the Ion Sequencing kit (Life Technologies). Bases callings were performed with Torrent Suite Software version 5.0.2. Mutations were obtained using Torrent Variant Caller using Somatic variant frequency and AD169_ATCC as reference. Mutations were then filtered against reference (Wild-type HCMV-BAC) using vcftools version 0.1.13.

### Viral immediate early and late protein expression

A transfection of mutated HCMV-BAC into human fibroblasts MRC-5 using liposomal reagent Transfast^TM^ (Promega, USA) was performed. Cells were fixed at 5 days post transfection, and immunostaining was performed for viral immediate early (anti-IE1 antibody; Argene, France) and late (anti-gB antibody; Abcam, United Kingdom) proteins in transfected cells.

### Plasmids construction for Alpha analysis

For protein production, the SC784 expression plasmid encoding full-length amino-terminal 3xHA-tagged pUL56 and driven by an upstream HCMV major immediate early promoter was cloned in vector pGEM3z. In-Fusion® (Clontech, USA) kit was used following manufacturer’s instructions to clone several *UL56* mutants from source HCMV-BAC in SC784 plasmid. ORF encoding pUL89 is composed of two exons separated by an intron. Both exons were generated by assembling PCR from AD169 strain and cloned into pCI-neo (Promega, USA) with His tag to obtain pCI-neo His-pUL89. Transformations were performed in DH5∝ cells. The nucleotide sequence of all constructs generated was verified by Sanger sequencing prior to use.

### Transfection and proteins purification

HEK293 were transfected with the appropriate expression vectors using liposomal reagent Transfast^TM^ following manufacturer’s instructions, washed and lysed 48 h later with CelLytic M (Sigma-Aldrich, USA). Lysates were cleared by centrifugation.

For purification of HA-tagged pUL56, the cell-free reaction was performed with Anti-HA Immunoprecipitation Kit according to the manufacturer’s protocol (Sigma-Aldrich, USA).

For purification of His-tagged pUL89, the cell-free reaction was performed with Ni resin (Clontech, USA).

All proteins were concentrated approximately 5-fold using Pall centrifugal filters (Pall, USA), and protein concentration was determined by the Bradford method using bovine serum albumin (Sigma-Aldrich, USA) as standard protein.

### Western blotting

SDS-PAGE was performed under reducing conditions on Mini-PROTEAN TGX Stain-Free gels (BioRad, USA). Proteins were then transferred onto a Trans-Blot Turbo PVDF Western blotting membrane (BioRad, USA). Antibody dilutions were 1:1,000 for the mouse anti-HA antibody (catalogue number: 2367, Cell Signaling, USA), mouse anti-His antibody (catalogue number: 2366, Cell Signaling, USA) and secondary anti-mouse horseradish peroxidase (HRP)-linked antibody (catalogue number: 7076, Cell Signaling, USA). Signals were visualized using the Substrat HRP Immobilon Western (Merck Millipore, USA) and a ChemiDoc imager (BioRad, USA).

### Protein/Protein interaction analysis by Alpha

Alpha (Amplified Luminescent Proximity Homogeneous Assay) experiments were conducted according to the manufacturer’s protocol (PerkinElmer, USA). Five μL of transfected MRC-5 lysate with HCMV-BAC is first disposed in wells of a 96-well AlphaPlate. The final concentration of each proteins was optimized to obtain the best value of interaction. Ten μL of each purified protein were combined (to give a final assay concentration of 500 nM of 3xHA-pUL56 and 1,5 µM of 6xHis-pUL89). Ten μL and 15 μL of 10 mg/mL of donor beads and acceptor beads, respectively, were added and incubated for 1 hour. Plates were read on a PerkinElmer EnVision^TM^ plate reader using an excitation wavelength of 680 nm and emission detection was set at 615 nm.

### Data availability

The datasets generated and/or analysed during the current study are available from the corresponding author on reasonable request.

## Electronic supplementary material

Supplementary materials

## References

[1] Alain S (2004). Detection of ganciclovir resistance after valacyclovir-prophylaxis in renal transplant recipients with active cytomegalovirus infection. J. Med. Virol..

[2] Hantz S (2010). Drug-resistant cytomegalovirus in transplant recipients: a French cohort study. J. Antimicrob. Chemother..

[3] Lischka P (2010). *In vitro* and *in vivo* activities of the novel anticytomegalovirus compound AIC246. Antimicrob. Agents Chemother..

[4] Melendez DP, Razonable RR (2015). Letermovir and inhibitors of the terminase complex: a promising new class of investigational antiviral drugs against human cytomegalovirus. Infect. Drug Resist.

[5] Borst EM, Wagner K, Binz A, Sodeik B, Messerle M (2008). The essential human cytomegalovirus gene UL52 is required for cleavage-packaging of the viral genome. J. Virol..

[6] Borst EM (2013). The human cytomegalovirus UL51 protein is essential for viral genome cleavage-packaging and interacts with the terminase subunits pUL56 and pUL89. J. Virol..

[7] Borst EM (2016). The Essential Human Cytomegalovirus Proteins pUL77 and pUL93 are Structural Components Necessary for Viral Genome Encapsidation. J. Virol.

[8] Köppen-Rung P, Dittmer A, Bogner E (2016). Intracellular distributions of capsid-associated pUL77 of HCMV and interactions with packaging proteins and pUL93. J. Virol.

[9] DeRussy BM, Tandon R (2015). Human cytomegalovirus pUL93 is required for viral genome cleavage and packaging. J. Virol.

[10] DeRussy BM, Boland MT, Tandon R (2016). Human Cytomegalovirus pUL93 Links Nucleocapsid Maturation and Nuclear Egress. J. Virol.

[11] Scholz B, Rechter S, Drach JC, Townsend LB, Bogner E (2003). Identification of the ATP-binding site in the terminase subunit pUL56 of human cytomegalovirus. Nucleic Acids Res.

[12] Hwang J-S, Bogner E (2002). ATPase activity of the terminase subunit pUL56 of human cytomegalovirus. J. Biol. Chem..

[13] Thoma C (2006). Identification of the interaction domain of the small terminase subunit pUL89 with the large subunit pUL56 of human cytomegalovirus. Biochemistry (Mosc.).

[14] Couvreux A (2010). Insight into the structure of the pUL89 C-terminal domain of the human cytomegalovirus terminase complex. Proteins.

[15] Nadal M (2010). Structure and inhibition of herpesvirus DNA packaging terminase nuclease domain. Proc. Natl. Acad. Sci. USA.

[16] Eilers M, Patel AB, Liu W, Smith SO (2002). Comparison of helix interactions in membrane and soluble alpha-bundle proteins. Biophys. J..

[17] Ansari S, Helms V (2005). Statistical analysis of predominantly transient protein-protein interfaces. Proteins.

[18] Scheffczik H, Savva CGW, Holzenburg A, Kolesnikova L, Bogner E (2002). The terminase subunits pUL56 and pUL89 of human cytomegalovirus are DNA-metabolizing proteins with toroidal structure. Nucleic Acids Res.

[19] Bradley AJ (2009). High-throughput sequence analysis of variants of human cytomegalovirus strains Towne and AD169. J. Gen. Virol..

[20] Ullman EF (1994). Luminescent oxygen channeling immunoassay: measurement of particle binding kinetics by chemiluminescence. Proc. Natl. Acad. Sci. U. S. A..

[21] Waller H, Chatterji U, Gallay P, Parkinson T, Targett-Adams P (2010). The use of AlphaLISA technology to detect interaction between hepatitis C virus-encoded NS5A and cyclophilin A. J. Virol. Methods.

[22] Champier G (2008). Putative functional domains of human cytomegalovirus pUL56 involved in dimerization and benzimidazole D-ribonucleoside activity. Antivir. Ther..

[23] Giesen K, Radsak K, Bogner E (2000). The potential terminase subunit of human cytomegalovirus, pUL56, is translocated into the nucleus by its own nuclear localization signal and interacts with importin alpha. J. Gen. Virol.

[24] Buerger I (2001). A novel nonnucleoside inhibitor specifically targets cytomegalovirus DNA maturation via the UL89 and UL56 gene products. J. Virol..

[25] Goldner T (2011). The novel anticytomegalovirus compound AIC246 (Letermovir) inhibits human cytomegalovirus replication through a specific antiviral mechanism that involves the viral terminase. J. Virol..

[26] Chou S (2015). Rapid *In Vitro* Evolution of Human Cytomegalovirus UL56 Mutations That Confer Letermovir Resistance. Antimicrob. Agents Chemother..

[27] Walker MP, Yao N, Hong Z (2003). Promising candidates for the treatment of chronic hepatitis C. Expert Opin. Investig. Drugs.

[28] Chee MS (1990). Analysis of the protein-coding content of the sequence of human cytomegalovirus strain AD169. Curr. Top. Microbiol. Immunol.

[29] Sievers F (2011). Fast, scalable generation of high-quality protein multiple sequence alignments using Clustal Omega. Mol. Syst. Biol..

[30] McWilliam H (2013). Analysis Tool Web Services from the EMBL-EBI. Nucleic Acids Res.

[31] Li W (2015). The EMBL-EBI bioinformatics web and programmatic tools framework. Nucleic Acids Res.

[32] Kelley LA, Mezulis S, Yates CM, Wass MN, Sternberg MJE (2015). The Phyre2 web portal for protein modeling, prediction and analysis. Nat. Protoc..

[33] Borst EM, Hahn G, Koszinowski UH, Messerle M (1999). Cloning of the human cytomegalovirus (HCMV) genome as an infectious bacterial artificial chromosome in Escherichia coli: a new approach for construction of HCMV mutants. J. Virol..

[34] Hantz S (2013). Novel DNA polymerase mutations conferring cytomegalovirus resistance: input of BAC-recombinant phenotyping and 3D model. Antiviral Res..

