# Supplementary material for: Identification of a short sequence in the HCMV terminase pUL56 essential for interaction with pUL89 subunit
Source: Sci Rep. 2017 Aug 18;7:8796. doi: 10.1038/s41598-017-09469-7 (PMC5562894; doi:10.1038/s41598-017-09469-7)
Supplement: Supplementary file 1 — Supplementary materials [file 41598_2017_9469_MOESM1_ESM.docx]

Supplementary Materials for

Identification of a short sequence in the HCMV terminase pUL56 essential for interaction with pUL89 subunit

G. Ligat^1^, C. Jacquet^1^, S. Chou^2^, A. Couvreux^1^, S. Alain^1^, S. Hantz^1^*

correspondence to: [sebastien.hantz@unilim.fr](mailto:xxxxx@xxxx.xxx)

| Identification | Sequence identification | Accession number | Reference |
| --- | --- | --- | --- |
| HSV_1 | Human herpesvirus type 1 pUL28 packaging protein | CAA32321 | Dolan et al., 1992 |
| HSV_2 | Human herpesvirus type 2 pUL28 packaging protein | CAB0675 | Barnett et al., 1992 |
| CeHV_1 | Cercopithecine herpesvirus 1 pUL28 | BAC58068 | Pelyrigina et al., 2003 |
| CeHV_2 | Cercopithecine herpesvirus 2 pUL28 | AAU84532 | Tyler, Peters, and Severini, 2005 |
| EHV_1 | Equine herpesvirus 1 ORF32 | YP_053077 | Telford et al., 1992 |
| GaHV_3 | Gallid herpesvirus 3 pUL28 | BAB16538 | Izumiya et al., 2001 |
| MeHV_1 | Meleagrid herpesvirus 1 pUL28 processing and transport protein | AAG30068 | Kingham et al., 2001 |
| HHV_3 | Human herpesvirus 3 (Varicella-Zooster virus) | CAA27913 | Davison and Scott, 1986 |
| SUID | Suid herpesvirus 1 (Pseudorabies virus) UL28 | YP_068331 | Klupp et al., 2004 |
| GaHV_1 | Gallid herpesvirus 1 UL28-like protein | YP_182357 | Thureen et al., 2006 |
| GaHV_2 | Gallid herpesvirus 2 Homologue of HSV 1 ICP 18.5 packaging protein | AAF66763 | Kato et al., 1999 |
| AD169 | Human cytomegalovirus AD169 strain pUL56 | X17403 | Chee et al., 1990 |
| HHV_6_B | Human herpesvirus 6 type B transport protein | Q9WT24 | Isegawa et al., 1999 |
| HHV_6_A_GS | Human herpesvirus 6 type A transport protein | AHK06969 | None |
| CCMV | Chimpanzee herpesvirus pUL56 | AAM00906 | Davison et al., 2003 |
| RhCMV | Cercopithecine herpesvirus 8 (Rhesus cytomegalovirus) putative pUL56 DNA packaging protein | AAZ80592 | None |
| MCMV | Murid herpesvirus 1 (Murine cytomegalovirus) major DNA-binding protein | CAA47415 | Messerle et al., 1992 |
| RCMV | Murid herpesvirus 2 (Rat cytomegalovirus Maastricht) pR56 protein | AAC56431 | Beuken et al., 1996 |
| HHV_8 | Human herpesvirus 8 BALF3 EBV homolog, transport protein homologue | AAC57084 | Moore et al., 1996 |
| HHV_4_1 | Human herpesvirus 4 type 1 BALF3 | YP_401715 | de Jesus et al., 2003 |
| HHV_4_2 | Human herpesvirus 4 type 2 BALF3 | YP001129509 | Dolan A et al., 2006 |

**Table S1.** Sequences used for alignment of pUL56 and homologues.

**Table S2.** Sequences of *UL56*-primers used for deletion or combinations of mutations.

| Mutant BAC | Primer direction | Primer sequence |
| --- | --- | --- |
| *UL56* Del W671 - F680 | Forward | TTTGGTCAAATGCGCGGAAGGTACCGTGTATCCCAGTGAGAATTTTTCGGACTGTCAGGATAGGGATAACAGGGTAATCGATTT |
|  | Reverse | CCTTCTGCAGCACGTTTAGGTCCTGACAGTCCGAAAAATTCTCACTGGGATACACGGTACGCCAGTGTTACAACCAATTAACC |
| *UL56* W671A Y676A F679A F680A | Forward | TTTGGTCAAATGCGCGGAAGGTACCGTGTATCCCAGTGAGGCGATGGTGGTGAAGGCTATGGGTGCTGCCAATTTTTCGGACTGTCAGGATAGGGATAACAGGGTAATCGATTT |
|  | Reverse | CCTTCTGCAGCACGTTTAGGTCCTGACAGTCCGAAAAATTGGCAGCACCCATAGCCTTCACCACCATCGCCTCACTGGGATACACGGTACGCCAGTGTTACAACCAATTAACC |
| *UL56* W671A | Forward | TTGGTCAAATGCGCGGAAGGTACCGTGTATCCCAGTGAGGCGATGGTGGTGAAGTATATGGGTAGGGATAACAGGGTAATCGATTT |
|  | Reverse | AGTCCGAAAAATTGAAAAAACCCATATACTTCACCACCATGGCCTCACTGGGATACACGGTACGCCAGTGTTACAACCAATTAACC |
| *UL56* Y676A | Forward | GGAAGGTACCGTGTATCCCAGTGAGTGGATGGTGGTGAAGGCTATGGGTTTTTTCAATTTTTCTAGGGATAACAGGGTAATCGATTT |
|  | Reverse | CGTTTAGGTCCTGACAGTCCGAAAAATTGAAAAAACCCATAGCCTTCACCACCATCCACTCACGCCAGTGTTACAACCAATTAACC |
| *UL56* F679A | Forward | CGTGTATCCCAGTGAGTGGATGGTGGTGAAGTATATGGGTGCTTTCAATTTTTCGGACTGTCATAGGGATAACAGGGTAATCGATTT |
|  | Reverse | TCTGCAGCACGTTTAGGTCCTGACAGTCCGAAAAATTGAAAGCACCCATATACTTCACCACCAGCCAGTGTTACAACCAATTAACC |
| *UL56* F680A | Forward | GTATCCCAGTGAGTGGATGGTGGTGAAGTATATGGGTTTTGCCAATTTTTCGGACTGTCAGGATAGGGATAACAGGGTAATCGATTT |
|  | Reverse | CCTTCTGCAGCACGTTTAGGTCCTGACAGTCCGAAAAATTGGCAAAACCCATATACTTCACCAGCCAGTGTTACAACCAATTAACC |
